# Modulatory effects of vagal stimulation on neurophysiological parameters and the cellular immune response in the rat brain during systemic inflammation

**DOI:** 10.1186/s40635-016-0091-4

**Published:** 2016-06-29

**Authors:** Hanna Schweighöfer, Christoph Rummel, Joachim Roth, Bernhard Rosengarten

**Affiliations:** Department of Neurology, Justus-Liebig-University Giessen, Klinikstr. 33, 35392 Giessen, Germany; Institute of Veterinary Physiology and Biochemistry, Justus-Liebig-University Giessen, 35392 Giessen, Germany

**Keywords:** Sepsis, Neurovascular coupling, Immune-to-brain signaling, Vagus nerve stimulation, Nuclear factor kappa B, Nuclear factor interleukin 6, Cytokines, Leptin, Prostaglandins

## Abstract

**Background:**

Stimulation of the vagus nerve has modulating, anti-inflammatory effects on the cellular immune response in the blood and the spleen, stabilizing brain function. Here, we aimed to investigate its potential effects on immune-to-brain communication focusing on neurophysiological readouts and leukocyte migration to the brain during severe sepsis-like endotoxemia.

**Methods:**

Systemic inflammation was induced by intravenous administration of lipopolysaccharide (LPS; 5 mg/kg). Animals received either no manipulation of the vagus nerve, vagotomy, or vagotomy plus vagus nerve stimulation of the distal trunk. Somatosensory evoked potentials and evoked flow velocity response were measured for 4.5 h as indicators of brain function and neurovascular coupling, respectively. In addition, brain areas with (cortex) and without (hypothalamus) tight blood-brain barrier were studied separately using immunohistochemistry and RT-PCR. Moreover, plasma cytokine and leptin levels were analyzed by ELISA.

**Results:**

LPS induced a decline of both neurophysiological parameters, which was prevented by vagus nerve stimulation. As for peripheral organs, LPS-stimulated neutrophil counts increased in the brain and colocalized in the brain with endothelial intercellular adhesion molecule (ICAM)-1. Interestingly, vagal stimulation reduced this colocalization and decreased nuclear translocation of the brain cell activation marker nuclear factor interleukin 6 (NF-IL6). Furthermore, it reduced the gene expression of inflammatory markers and extravasation signals (IL-6, CXCL-1, ICAM-1) in the hypothalamus but not the cortex linked to a moderate decrease in circulating cytokine levels (interleukin 6, tumor necrosis factor alpha) as well as lower plasma leptin concentration.

**Conclusions:**

Our data suggest beneficial effects of anti-inflammatory vagus nerve stimulation on brain function by reducing the interaction of neurotrophil granulocytes with the brain endothelium as well as attenuating inflammatory responses in brain areas lacking a blood-brain barrier.

**Electronic supplementary material:**

The online version of this article (doi:10.1186/s40635-016-0091-4) contains supplementary material, which is available to authorized users.

## Background

Systemic inflammation results from host-related activation of innate immunity in response to infectious or non-infectious stimuli. In case of dysregulation of the inflammatory response, this naturally occurring process may bear deleterious effects for the host and frequently results in tissue injury, organ failure, and even mortality [1, 2]. In recent years, many steps and key players in the pathway of immune activation have been described. It became evident that the initial systemic inflammatory response syndrome (SIRS) in general exceeds the needs for antimicrobial defense leading to the concept of host-related hyperinflammatory organ dysfunction as a relevant “motor” of developing sepsis [3]. Consequently, anti-inflammatory intervention strategies such as anti-cytokine antibody therapies have been developed, which interfere with the early steps of the inflammatory cascade. Whereas they were highly effective in experimental conditions, they failed clinically because manifest clinical symptoms in humans mostly occur at the later stages of the inflammatory response when the agents already lost their therapeutic relevance [4–6].

Nowadays, research concentrates on endogenous protection systems and aims to test their clinical feasibility. As such, vagus nerve stimulation is a prospective candidate for neuromodulation: several reports demonstrate a clinically relevant anti-inflammatory effect due to the stimulation of the efferent fibers of the vagus nerve. The efferent fibers originate from the nucleus (*Ncl.*) *dorsalis vagi* and represent the cranial parasympathetic autonomic nervous system innervating the heart, lung, and the oro-intestinal tract until the Cannon Böhm point at the left colon flexure. The efferent pathway of the anti-inflammatory vagus nerve reflex is still not fully understood but there are many reports showing a multisynaptically mediated functional influence on spleen lymphocytes involving the alpha7-nicotinic receptor [7]. However, the vagus also consists of afferent fibers terminating in the *Ncl. tractus solitarii*. The *Ncl. tractus solitarius* is linked to many sites in the brain affecting sickness behavior (several brain structures involved), temperature homeostasis (hypothalamus), the hormonal stress response (hypothalamus, pituitary), and the central sympathetic nervous system (*Ncl. coeruleus*) [8]. Furthermore, afferent fibers can induce cortical inflammation [9, 10]. Due to the latter effect, most studies perform efferent vagus nerve stimulation by dissecting the nervus (*N*.) *vagus* and stimulating the distal part of the nerve trunk electrically [11].

Microcirculatory dysfunction is a key player of inflammation-related side effects leading to inappropriate blood supply and, therefore, functional failure of active organ cells. One of the most sensitive organs is the brain, which depends on continuous and appropriate blood supply because of its high energy demands and lack of stores for oxygen and/or energy substrates. Indeed, sepsis-related delirium is a frequent and early finding, which even precedes diagnosis of sepsis and appears in up to two thirds of sepsis patients [12].

In the present study, we aimed to extend our previous research on vagal anti-inflammatory properties [13, 14] to its effects on brain regions with (cortex) or without (hypothalamus) a tight blood-brain barrier. Therefore, several immune-to-brain signaling pathways (humoral and cellular) and different target structures related to the neurovascular coupling mechanism were studies in this well-established endotoxic lipopolysaccharide (LPS) sepsis model.

## Methods

### General animal preparation

Adult male Sprague Dawley rats (weighing 290 to 350 g) were purchased from Charles River (Sulzfeld, Germany). They were maintained on a 12-h light/dark cycle and housed four to a cage with food and water available ad libitum. Initially, rats were anesthetized with 1.5 to 3 % isoflurane, tracheotomized, paralyzed with pancuronium bromide (0.2 mg/kg/h; Inresa Arzneimittel GmbH, Freiburg, Germany) and mechanically ventilated (Harvard Rodent Ventilator; Harvard Apparatus, South Natick, MA, USA) with a 1:1 mixture of nitrogen and oxygen. For recording of blood pressure, blood sampling, and drug administration, the right femoral artery and vein were cannulated. Isoflurane anesthesia was then replaced by an intravenous application of an α-chloralose bolus (60 mg/kg; Sigma-Aldrich Chemie GmbH, Taufkirchen, Germany) and maintained by a continuous administration of α-chloralose (40 mg/kg/h). A washout period of 60 min was maintained before the neurophysiological measurements were started. Arterial blood gas analysis and pH (blood gas analyzer model Rapidlab 348; Bayer Vital GmbH, Fernwald, Germany) as well as glucose (Glukometer Elite XL, Bayer Vital GmbH, Fernwald, Germany) and lactate (Arkray Inc. European Office, Düsseldorf, Germany) were measured at least every 60 min. To replace renal and perspirative fluid losses, a moderate volume therapy of 1.2 ml/h of 0.9 % NaCl was provided. Additionally, glucose concentrations in the blood were maintained at >60 mg/dl and body core temperature was maintained at 37 °C using a heating pad.

All animal experiments were conducted in strict accordance with the National Institutes of Health Guide for Care and Use of Laboratory Animals and approved by the local ethics committee (ethics approval number GI 20/18–81/2011).

### Neurovascular coupling measurement

The head of the animals was placed in a stereotaxic frame and the apex of the skull was exposed. To allow transcranial laser-Doppler flowmetry (LDF), the bone over the left parietal cortex was thinned with a saline-cooled drill (Dremel Moto-flex, Dremel Europe, Breda, The Netherlands) [15]. In accordance with the coordinates of the somatosensory cortex, the laser probe (BRL-100, Harvard Apparatus, Holliston, MA, USA) was placed 3.5 mm lateral and 1 mm rostral to bregma; this corresponds to the region of maximal hemodynamic response during contralateral forepaw stimulation [16–18]. For the continuous recording and processing of the laser-Doppler signal and mean arterial blood pressure, a data acquisition software (Neurodyn, HSE, March-Hugstetten, Germany) was used.

Electric brain activity was measured monopolarily with one active calomel electrode about 0.5 mm behind the laser probe and one indifferent calomel electrode located on the nasal bone. Signals were recorded and amplified (BPA Module 675, HSE, March-Hugstetten, Germany) and in the following somatosensory evoked potentials (SEP) were averaged (Neurodyn acquisition software, HSE, March-Hugstetten, Germany). To calculate the SEP amplitudes, the difference between N2 and P1 was used.

To achieve somatosensory activation, rectangular bipolar pulses of 1.5 mA, 0.3 ms, and 2 Hz were applied by small needle electrodes placed under the skin of the right forepaw (PSM Module 676; HSE, March-Hugstetten, Germany). Stimulation with 1.5 mA ensured that there were no pain-associated changes in systemic blood pressure [16–18]. Electric stimulation was carried out for 30 s, followed by 30 s of non-stimulation. These activation-rest cycles were performed ten times to increase the signal to noise ratio.

Whereas the Doppler does not measure absolute values, signal changes closely correlate with flow changes [16, 17]. Therefore, flow velocity responses were averaged and the relative responses were calculated in relation to the resting phase, which was set to zero. The evoked flow velocity responses (EFVR) were then calculated from the averaged relative flow velocity signals under conditions of stimulation [13].

### Vagus nerve stimulation

Both vagus nerves were exposed at the cervical level and carefully dissected from the common carotid artery in each rat. Animals received either no further manipulation of the vagus nerve (sham surgery (SHAM) and SHAM + LPS), a bilateral vagotomy (LPS + VGX), or a bilateral vagotomy and a stimulation of the distal trunk of the left nervus vagus (LPS + VGX + VNS) with a special nerve stimulation clamp (HSE, March-Hugstetten, Germany). For electrical stimulation, pulses of 2 mA, 0.3 ms, and 2 Hz were applied 15 min before administration of LPS and during the time course of the experiment and were only interrupted during the neurophysiological measurements [11].

### Study design

Rats were assigned to the abovementioned groups in random order with 15 rats per group. Animals received 5 mg/kg body weight intravenous LPS (LPS from *Escherichia coli*, O111:B4; Sigma-Aldrich Chemie GmbH, Germany) resolved in 0.9 % NaCl or 0.5 ml of 0.9 % NaCl. LPS was administered slowly for 5 min. Experiments ended 4.5 h after LPS/vehicle administration, plasma samples were obtained, and brains were removed and stored at −80 °C. The reason for limiting the study to 4.5 h after LPS application was that the blood-brain barrier and the macrocirculation were still in the normal range [19].

### Tissue processing

Coronal 20 μm brain sections at the level of the subfornical organ (SFO, bregma −0.8 to −1.6 mm) were cut using a cryostat (2800 Grigocut E, Reichert-Jung, Nußloch, Germany). Brain sections were then thaw-mounted on poly-l-lysin-coated glass-slides and stored at −55 °C for immunohistochemistry. Additionally, brain sections between the vascular organ of the lamina terminalis and the median eminence (bregma 0.6 to −2.8) were stacked on glass-slides, and the hypothalamus was dissected. The cortex and the hypothalamus were divided into two pieces each (left and right hemisphere) and separately stored at −55 °C for polymerase chain reaction (PCR) analysis. Brain structures were identified using “The rat brain in stereotaxic coordinates” [20].

### Immunohistochemistry

Frozen brain sections were briefly air-dried at room temperature and fixed in 2 % paraformaldehyde (Merck, Darmstadt, Germany) diluted in phosphate-buffered saline (PBS) for 10 min. The sections were then washed three times in PBS and incubated in a blocking solution containing PBS, 10 % normal donkey serum (NDS, Biozol, Eching, Germany), and 0.1 % Triton X-100 (Sigma-Aldrich Chemie GmbH, Steinheim, Germany) for 1 h at room temperature. Subsequently, sections were incubated with the primary antibodies diluted in the blocking solution (rabbit-anti-myeloperoxidase (MPO), dilution 1:400, A 0398, Dako GmbH, Hamburg, Germany; goat-anti-intercellular adhesion molecule (ICAM)-1, dilution 1:1000, AF583, R&D Systems Inc, Minneapolis, MN, USA; goat-anti-nuclear factor interleukin 6 (NF-IL6), dilution 1:500, Sc-150-G, Santa Cruz Biotechnology, Santa Cruz, CA, USA) for 20–22 h at 4 °C. For the double labeling of neutrophil granulocytes and ICAM-1, the two antibodies were mixed in one solution. After washing three times in PBS, the sections were incubated for 2 h at room temperature with the secondary antibodies diluted in blocking solution (Alexa488-conjugated anti-rabbit IgG, dilution 1:500, AZA21206, MoBiTec GmbH, Göttingen, Germany; Cy3-conjugated anti-goat, dilution 1:600, 705-165-147, Jackson Immuno Research Laboratories, Inc., West Grove, PA, USA). Following another three washing steps, cell nuclei were counterstained with 4.6-diamidino-2-phenylindole (DAPI, dilution 1:8000 in PBS, Mobitec GmbH, Göttingen, Germany) for 10 min. Finally, sections were washed three times in PBS, cover slipped using Citifluor (Citifluor Ltd., London, UK) and stored at 4 °C until analysis.

Specificity of the used antibodies has previously been tested [21]. Moreover, control experiments by substitution of the primary antibodies with non-immunized animals IgG were performed.

### Microscopic analysis

A light/fluorescent Olympus BX50 microscope (Olympus Optical, Hamburg, Germany) and a black and white Spot Insight camera (Diagnostic Instruments, Visitron Systems, Puchheim, Germany) were used to acquire images from the stained sections. For each staining and time point, microphotographs were taken with the same exposure times using MetaMorph 7.7.5.0 software (Molecular Devices Inc., Downingtown, PA, USA). For NF-IL6 and the colocalization between neutrophil granulocytes and ICAM-1, the images were combined to RGB color images with help of the MetaMorph 5.05 software. All images were optimized for brightness and contrast using Adobe Photoshop 6.0 to the exact same extend within one analysis to preserve comparability (Adobe Systems Incorporated, San Jose, CA, USA).

### Quantification

For the quantification of nuclear NF-IL6 immunoreactivity, the DAPI-stained nuclei were labeled with “1” and counted on microphotographs with a ×100 magnification. Subsequently, NF-IL6-positive nuclei were labeled with “2”, counted, and given as percentage of all stained nuclei.

For the quantification of neutrophil granulocytes and ICAM-1, Fiji (Fiji Is Just ImageJ) software 2014 (open source software based on ImageJ modified by BioVoxxel, Mutterstadt, Germany) was used. After a median filter was applied to improve image noise, a threshold was set and the watershed function was used. Then, all particles over 20 pixels were automatically counted. For the number of neutrophil granulocytes, the number of particles on microphotographs with a ×5 and ×10 magnification and for the ICAM-1 immunoreactivity the percentage of the stained area on microphotographs with a ×20 magnification was used as readout.

The colocalization of neutrophil granulocytes and ICAM-1 was determined similar to the nuclear NF-IL6 immunoreactivity with a labeling of all neutrophil granulocytes and those that were colocalized with ICAM-1 on microphotographs with a ×10 magnification. The number of colocalized neutrophil granulocytes is given as percentage of all neutrophil granulocytes.

All images were processed in the same way, to guarantee comparability.

For all quantifications, three sections of all animals and five animals of each group were used. The mean of the three sections was taken for calculating the mean of each group.

Brain maps for overviews were modified from the digital version of “The rat brain in stereotaxic coordinates” [20] using CorelDraw 9 (Corel Corporation, Ottawa, Canada).

### Real-time PCR

Total ribonucleic acid (RNA) of the collected frozen cortex and hypothalamic sections (approximately 40 to 50 mg tissue) was extracted using Trizol (Invitrogen, Carlsbad, CA, USA) according to the manufacturer’s protocol. Reverse transcription of 1 μg total RNA was performed using 50 U murine leukemia virus reverse transcriptase, 50 μM random hexamers, and 10 mM deoxynucleoside triphosphates (dNTP) mix (Applied Biosystems, Foster City, CA, USA) in a total reaction volume of 20 μl. Afterwards, quantitative real-time PCR was performed in duplicate with preoptimized primer/probe mixture (TaqMan Gene Expression Assay, Applied Biosystems, Foster City, CA, USA) and TaqMan universal PCR Master Mix (Applied Biosystems, Foster City, CA, USA) on a StepOnePlus Real-Time PCR System (Applied Biosystems, Foster City, CA, USA). The following cycling protocol was used: polymerase activation, 50 °C for 2 min, denaturation, 95 °C for 10 min, and 35 to 44 cycles of 15 s denaturation at 95 °C followed by 1 min of annealing and elongation at 60 °C.

The cDNA quantities were normalized by measurement of a housekeeping gene as a reference. Out of the six tested genes (Double-dye (Hydrolysis) probe geNorm 6 gene kit, ge-DD-6, Primer Design Ltd, Southampton UK), β actin (4352340E, Applied Biosystems, Foster City, CA, USA) was chosen as the best housekeeping gene using DataAssist 3.01 (Thermo Fisher Scientific Inc., Waltham, MA, USA) and NormFinder version 4 (Department of Molecular Medicine (MOMA) Aarhus University Hospital, Aarhus, Denmark).

Using the ΔΔCT method, sample values were calculated as x-fold difference from a control sample (SHAM, value determined as 1) within the same experiment. Samples from the same five animals of each group that were used for immunohistochemistry were also used for PCR analysis.

The following gene expression assays from Applied Biosystems were used: ICAM-1 Rn00564227_m1, NF-IL6 Rn00824635_s1, IkBα Rn01473658_g1, SOCS3 Rn00585674_s1, IL-1β Rn00580432_m1, IL-6 Rn01410330_m1, IL-10 Rn99999012_m1, IL-17 Rn01757168, TNFα Rn99999017_m1, Elane (neutrophil elastase) Rn01535456_g1, CD168 Rn01495634, CXCL1 Rn00578225_m1, COX2 Rn00568225_m1, and mPGES1 Rn00572047ml.

### ELISA

Blood samples were drawn into tubes with heparin sodium 2500 (Ratiopharm, Ulm, Germany) at the end of the experiment. They were immediately centrifuged and separated, and plasma was stored at −80 °C. Tumor necrosis factor (TNF)α (Rat TNF ELISA Kit, 560479, BD Bioscience, San Diego, CA, USA), interleukin (IL)-6 (Rat IL-6 ELISA Kit, 550319, BD Bioscience, San Diego, CA, USA), IL-10 (Rat IL-10 ELISA Kit, 555134, BD Bioscience, San Diego, CA, USA), and leptin (Rat Leptin ELISA, EZRL-83K, Merck KgaA Millipore, Darmstadt, Germany) plasma levels were measured using enzyme-linked immunosorbent assays according to the manufacturer’s protocol.

The detection limits for the assays were 31.3 pg TNFα/ml, 78 pg IL-6/ml, 15.6 pg IL-10/ml, and 0.2 ng leptin/ml, respectively. For TNFα, IL-6, and IL-10 measurements, 8 samples of the SHAM group and 13 samples of all septic groups were used, and for leptin, 5 samples of the SHAM group and 8 samples of all septic groups were used.

### Statistics

Statistical analyses were performed using a one-way analysis of variance. In cases of significance, a Fisher post hoc test was applied (Statview, SAS, Cary, NA, USA). The SEP and EFVR data were analyzed separately for each time point. The significance level was set to *P* < 0.05. All data are presented as means ± standard error of the mean (SEM) or as means ± standard deviation (SD), respectively.

## Results

### Clinical and neurophysiological results and systemic cytokine responses

Due to the α-chloralose-induced anesthesia, all animals developed a metabolic alkalosis and according to the volume administration a slight decrease in hematocrit at the end of the experiment. Using a 1:1 oxygen/nitrogen gas caused a supraphysiological partial pressure of oxygen in all groups while the partial pressure of carbon dioxide due to controlled ventilation remained in the physiological range. The glucose plasma concentration was kept in the physiological range during the 4.5 h of the experiment. Animals of the SHAM group showed stable glucose, lactate, blood gas, hemodynamic, and neurophysiological parameters during the time course of the experiment. LPS-treated animals developed a typical and reproducible severe sepsis syndrome: the blood pressure and pH decreased significantly, whereas the lactate plasma levels were induced. Septic animals also developed a decline of the SEP amplitudes and the evoked flow velocity responses. In line with previous reports [13], vagus nerve stimulation mitigated effects on blood pressure, pH, and lactate levels and stabilized neurophysiological data (Tables 1 and 2).

**Table 1:** Group-averaged data for blood glucose, lactate, arterial pH, pO_2_, and pCO_2_

	Glucose (mg/dl)		Lactate (mmol/l)		pH		pO_2_ (mmHg)		pCO_2_ (mmHg)	
	Baseline	End	Baseline	End	Baseline	End	Baseline	End	Baseline	End
SHAM	82 ± 27	71 ± 13	1.3 ± 0.7	1.0 ± 1.0	7.50 ± 0.03	7.49 ± 0.04	252 ± 26	223 ± 16	35 ± 4	35 ± 2
SHAM + LPS	84 ± 26	72 ± 18	1.2 ± 0.6	5.1 ± 1.3***	7.49 ± 0.05	7.42 ± 0.04***	239 ± 16	214 ± 17	37 ± 3	36 ± 3
LPS + VGX	98 ± 20	75 ± 17	1.2 ± 0.7	4.9 ± 1.5***	7.48 ± 0.03	7.42 ± 0.05***	249 ± 17	223 ± 14	38 ± 4	34 ± 2
LPS + VGX + VNS	95 ± 24	79 ± 22	1.2 ± 0.5	3.9 ± 0.9***^#$^	7.49 ± 0.03	7.43 ± 0.04***	242 ± 24	220 ± 26	36 ± 5	36 ± 3^$^

Data are given as means ± standard deviation (SD) (*n* = 15) together with statistical differences. Significance is given as: ***P < 0.001; # compared with SHAM + LPS, ^#^ *P* < 0.05; $ compared with LPS + VGX, ^$^ *P* < 0.05

*LPS* lipopolysaccharide, *pO*_*2*_ partial pressure of oxygen, *pCO*_*2*_ partial pressure of carbon dioxide, *SHAM* sham surgery, *VGX* bilateral vagotomy, *VGX + VNS* bilateral vagotomy and distal vagus nerve stimulation

**Table 2:** Group-averaged data for hematocrit, mean arterial blood pressure, somatosensory evoked potentials, P1 latencies, and the evoked flow velocity responses

	Hematocrit		Mean BP (mmHg)		SEP (μV)		P1 latency (ms)		EFVR (%)	
	Baseline	End	Baseline	End	Baseline	End	Baseline	End	Baseline	End
SHAM	41 ± 3	36 ± 4	114 ± 12	100 ± 24	11 ± 5	10 ± 6	11.2 ± 0.9	11.9 ± 1.0	24 ± 6	20 ± 7
SHAM + LPS	40 ± 2	37 ± 1	107 ± 9	50 ± 12***	10 ± 4	4 ± 2***	11.6 ± 1.0	13.0 ± 1.4*	29 ± 11	10 ± 10**
LPS + VGX	42 ± 3	37 ± 2	108 ± 10	49 ± 13***	9 ± 4	4 ± 3***	11.6 ± 0.9	13.0 ± 1.4*	30 ± 9	12 ± 14*
LPS + VGX + VNS	42 ± 2	36 ± 1	114 ± 12	59 ± 19***	12 ± 5	7 ± 5*^#$^	11.5 ± 1.0	13.1 ± 1.4*	24 ± 9	19 ± 9^#$^

Data are given as means ± standard deviation (SD) (*n* = 15) together with statistical differences. Significance is given as: * compared with SHAM, *P < 0.05; **P < 0.01; ***P < 0.001; # compared with SHAM + LPS, ^#^ *P* < 0.05; $ compared with LPS + VGX, ^$^ *P* < 0.05

*BP* blood pressure, *EFVR* evoked flow velocity responses, *LPS* lipopolysaccharide, *SEP* somatosensory evoked potentials, *SHAM* sham surgery, *VGX* bilateral vagotomy, *VGX + STIM* bilateral vagotomy and distal vagus nerve stimulation

Regarding systemic cytokine levels, vagus nerve stimulation but not vagotomy significantly reduced TNFα and IL-6 levels in LPS-treated groups as compared to the SHAM + LPS group (Table 3).

**Table 3:** Group-averaged plasma concentration of TNFα, IL-10 in pg/ml and IL-6 in ng/ml

	TNFα (ng/ml)	IL-6 (ng/ml)	IL-10 (ng/ml)
SHAM	0.275 ± 0.080	2.15 ± 0.44	0.834 ± 0.260
SHAM + LPS	3.461 ± 0.645***	1251 ± 250***	5.619 ± 0.759***
LPS + VGX	3.384 ± 0.501***	916 ± 143***	7.642 ± 0.547***^$^
LPS + VGX + VNS	2.177 ± 0.193*^#^	549 ± 68**^#^	5.489 ± 0.514***

Data are given as means ± standard error (SEM) together with statistical differences. Significance is given as: * compared with SHAM, *P < 0.05; **P < 0.01; ***P < 0.001; # compared with SHAM + LPS, ^#^ *P* < 0.05; $ compared with LPS + VGX, ^$^ *P* < 0.05. TNFα: SHAM, *n* = 8, SHAM + LPS, LPS + VGX + VNS, *n* = 12, LPS + VGX, *n* = 13; IL-6, IL-10: SHAM, *n* = 8, SHAM + LPS, LPS + VGX, LPS + VGX + VNS, *n* = 13

*TNF* tumor necrosis factor, *IL* interleukin, *SHAM* sham surgery, *VGX* bilateral vagotomy, *VGX + VNS* bilateral vagotomy and distal vagus nerve stimulation

### Cytokine expression in the hypothalamus and cortex

**Fig. 1:**
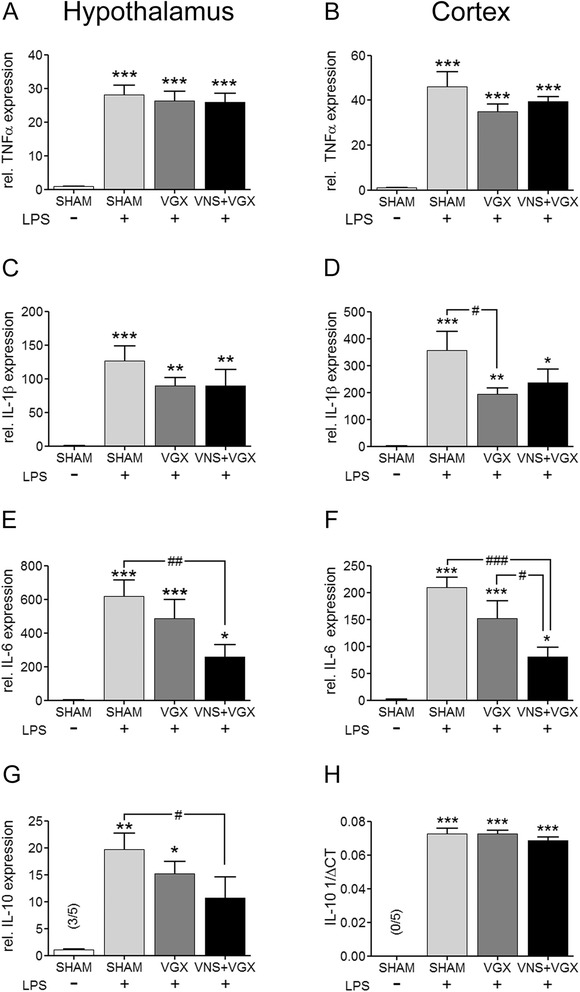
Real-time PCR analysis of cytokines in the hypothalamus (**a**, **c**, **e**, **g**) and in the cortex (**b**, **d**, **f**, **h**) 4.5 h after LPS or vehicle administration (*n* = 5 for each group). LPS induced an increased expression of TNFα (**a**, **b**), IL-1β (**c**, **d**), IL-6 (**e**, **f**), and IL-10 (**g**, **h**). Vagus nerve stimulation significantly reduced the increased expression of IL-6 in the hypothalamus and cortex compared to the LPS + SHAM group (*P* < 0.01) and the LPS + SHAM group (*P* < 0.001) and the LPS + VGX group (*P* < 0.05), respectively. Additionally, it reduced the IL-10 expression in the hypothalamus compared to the LPS + SHAM group (*P* < 0.05). However, it did not influence the TNFα or IL-1β expression. Vagotomy alone reduced the expression of IL-1β in the cortex compared to the LPS + Sham group (*P* < 0.05). Data are given as means ± SEM. Significance is given as: *compared to SHAM; *P < 0.05; **P < 0.01; ***P < 0.001; ^#^compared as indicated; ^#^ *P* < 0.05; ^##^ *P* < 0.01; ^###^ *P* < 0.001; *TNF* tumor necrosis factor, *IL* interleukin, *LPS* lipopolysaccharide, *SHAM* sham surgery, *VGX* bilateral vagotomy, *VGX + VNS* bilateral vagotomy and distal vagus nerve stimulation

Intravenous LPS application induced messenger RNA (mRNA) expression of TNFα, IL-1β, IL-6 and IL-10 in the cortex and hypothalamus of rats after 4.5 h (Fig. 1). Vagus nerve stimulation but not vagotomy significantly reduced the LPS-induced expression of IL-6 in the hypothalamus and cortex compared to the LPS + SHAM group. Vagal stimulation also decreased the IL-10 mRNA expression in the hypothalamus. Interestingly, vagotomy caused a significant reduction of the IL-1β mRNA expression in the cortex, whereas vagus nerve stimulation showed only a trend to a decline in the IL-1β mRNA expression (*P* = 0.07).

### Effects on intracellular signal molecules of inflammation

**Fig. 2:**
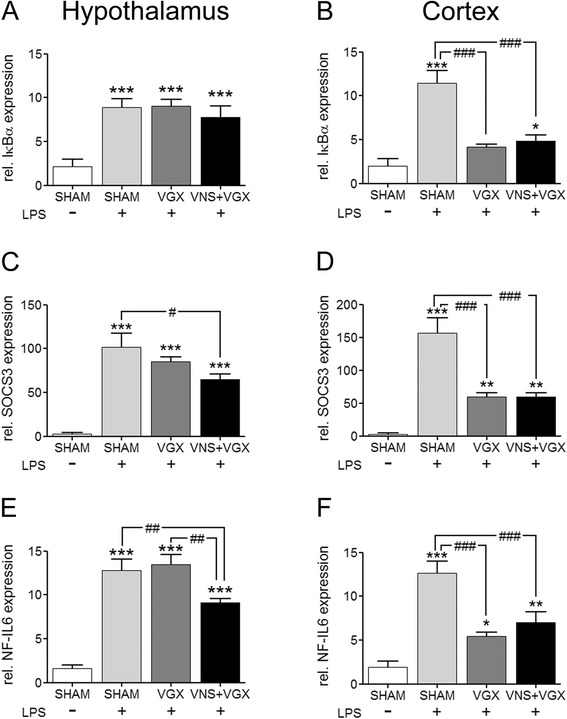
Real-time PCR analysis of IkBα (**a**, **b**), SOCS3 (**c**, **d**), and NF-IL6 (**e**, **f**) in the hypothalamus (**a**, **c**, **e**) and in the cortex (**b**, **d**, **f**) 4.5 h after LPS or vehicle administration (*n* = 5). LPS caused an increase of the analyzed mediators in the hypothalamus and cortex. In the hypothalamus, vagus nerve stimulation significantly reduced the SOCS3 expression compared to the LPS + SHAM group (*P* < 0.05) and the NF-IL6 expression compared to the LPS + SHAM group (*P* < 0.01) and the LPS + VGX group (*P* < 0.01). Interestingly, in the cortex, vagotomy alone, independent of vagus nerve stimulation, caused a highly significant decrease of the IkBα and SOCS3 as well as NF-IL6 (*P* < 0.001) expression. Data are given as means ± SEM. Significance is given as: * compared to SHAM; *P < 0.05; **P < 0.01; ***P < 0.001; # compared as indicated; ^#^ *P* < 0.05; ^##^ *P* < 0.01; ^###^ *P* < 0.001; *IkBα* inhibitor of kBα, *SOCS* suppressor of cytokine signaling, *NF-IL6* nuclear factor IL-6, *LPS* lipopolysaccharide, *SHAM* sham surgery, *VGX* bilateral vagotomy, *VGX + VNS* bilateral vagotomy and distal vagus nerve stimulation

**Fig. 3:**
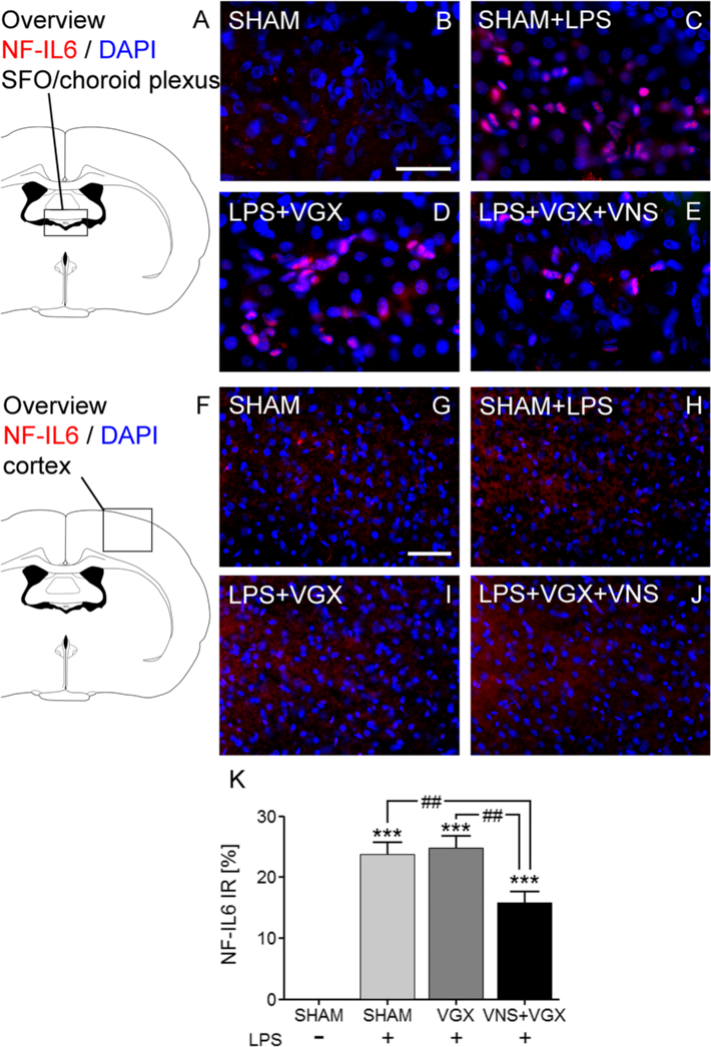
Immunohistochemical analysis of NF-IL6 in the SFO and neighboring choroid plexus (**a**–**e**) and in the cortex (**f**–**j**) 4.5 h after LPS or vehicle administration (*n* = 5). In the hypothalamus, there was no nuclear NF-IL6 immunoreactivity in the SHAM group (**b**) while all septic groups (**c**–**e**) showed strong intranuclear NF-IL6 signals. Vagus nerve stimulation (**e**) significantly reduced the percentage of nuclear NF-IL6 signals compared to the LPS + SHAM group (*P* < 0.01) and the LPS + VGX group (*P* < 0.01) (**k**). In the cortex, hardly any nuclear NF-IL6 signals could be detected after 4.5 h (**g**–**j**). The scale bar in picture B represents 25 μm and applies to pictures B–E and the scale bar in picture G represents 50 μm and applies to pictures **g**–**j**. Cell nuclei are stained in *blue* and NF-IL6 in *red*. Data are given as means ± SEM. Significance is given as: * compared to SHAM; *P < 0.05; **P < 0.01; ***P < 0.001; # compared as indicated; ^#^ *P* < 0.05; ^##^ *P* < 0.01; ^###^ *P* < 0.001; *NF-IL6* nuclear factor IL-6, *SFO* subfornical organ, *LPS* lipopolysaccharide, *SHAM* sham surgery, *VGX* bilateral vagotomy, *VGX + VNS* bilateral vagotomy and distal vagus nerve stimulation

LPS increased the expression of the inhibitor of kBα (IkBα), the suppressor of cytokine signaling (SOCS)3, and the nuclear factor interleukin (NF-IL)6 in the hypothalamus and the cortex (Fig. 2). In the hypothalamus, vagus nerve stimulation significantly inhibited the expression of SOCS3 and NF-IL6 mRNA expression as well as NF-IL6 immunoreactivity in the SFO and neighboring choroid plexus (Fig. 3). In the cortex, vagus nerve stimulation significantly attenuated the LPS-induced expression of IkBα and SOCS3 as well as NF-IL6. Interestingly, and only in the cortex, also, vagotomy blocked the increased expression of IkBα and SOCS3 as well as NF-IL6.

### Vagus nerve stimulation reduces COX2 expression in the hypothalamus, and vagotomy reduces COX2 expression and microglial activation in the cortex

**Fig. 4:**
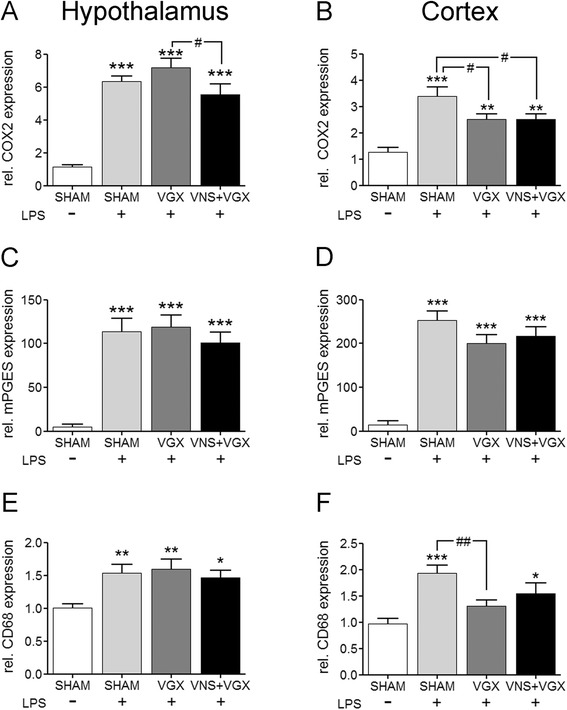
Real-time PCR analysis of COX2 (**a**, **b**), mPGES-1 (**b**, **d**), and the microglial activation marker CD68 in the hypothalamus (**a**, **c**, **e**) and in the cortex (**b**, **d**, **f**) (*n* = 5). COX2 and mPGES-1 expression was induced 4.5 h after LPS administration in the cortex as well as in the hypothalamus. The mPGES-1 expression did not differ significantly between the septic groups. In the hypothalamus, vagotomy increased and vagus nerve stimulation decreased COX2 expression resulting in significant differences (*P* < 0.05) whereas in the cortex both interventions resulted in a significant decrease in expression (both *P* < 0.05). Moreover, LPS induced the expression of CD68 in the hypothalamus and cortex. While there were no differences between the septic groups in the hypothalamus, vagotomy leads to a reduced CD68 expression in the cortex compared to the LPS + SHAM group (*P* < 0.01). Data are given as means ± SEM. Significance is given as: * compared to SHAM; *P < 0.05; **P < 0.01; ***P < 0.001; # compared as indicated; ^#^ *P* < 0.05; ^##^ *P* < 0.01; *COX* cyclooxygenase, *mPGES* microsomal prostaglandin synthase, *LPS* lipopolysaccharide, *SHAM* sham surgery, *VGX* bilateral vagotomy, *VGX + VNS* bilateral vagotomy and distal vagus nerve stimulation

LPS induced the expression of the inducible rate-limiting enzymes of the prostaglandin E2 (PGE2) synthesis cyclooxygenase 2 (COX2) and microsomal prostaglandin E synthase (mPGES)-1 (Fig. 4). There were no differences in the expression of mPGES-1 between the septic groups. COX2 expression was reduced in the cortex in the vagus nerve stimulated as well as vagotomized group. In the hypothalamus, vagotomy slightly increased the LPS-induced COX2 expression, whereas the vagus nerve stimulation reduced the expression resulting in a significant difference between both intervention groups.

In addition, LPS induced the expression of the microglial activation marker CD68 in the hypothalamus and in the cortex. There were no differences in the CD68 expression between the septic groups in the hypothalamus. In the cortex, vagotomy lead to a reduced CD68 expression compared to the LPS + SHAM group.

### Vagal effects on chemokines and adhesion molecules in the brain

**Fig. 5:**
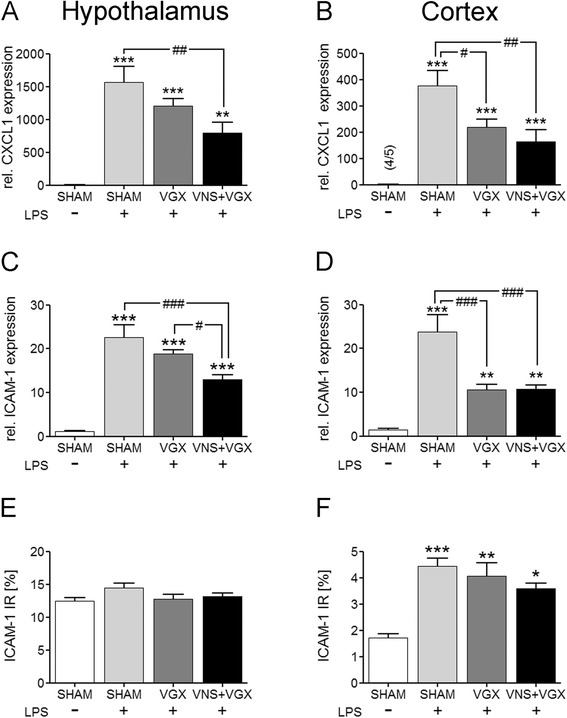
Real-time PCR analysis of CXCL1 (**a**, **b**) and ICAM-1 (**c**, **d**) in the hypothalamus (**a**, **c**) and cortex (**b**, **d**) as well as the quantification of immunohistochemical analysis of ICAM-1 in the SFO and neighboring choroid plexus (**e**) and in the cortex (**f**) (*n* = 5). CXCL1 and ICAM-1 expression was induced 4.5 h after LPS administration. In the hypothalamus, vagus nerve stimulation leads to a decreased expression of CXCL1 compared to the LPS + SHAM group (*P* < 0.01) and a decreased expression of ICAM-1 compared to the LPS + SHAM group (*P* < 0.01) and the LPS + VGX group (*P* < 0.05). The immunohistochemical analysis of the SFO and neighboring choroid plexus revealed no differences between the different groups. In contrast to the hypothalamus, in the cortex, vagotomy independent of vagus nerve stimulation caused a decreased expression of CXCL1 (*P* < 0.05 and *P* < 0.01) and ICAM-1 (*P* < 0.001) compared to the LPS + SHAM group, although this did not result in immunohistologically significant differences. Data are given as means ± SEM. Significance is given as: * compared to SHAM; *P < 0.05; **P < 0.01; ***P < 0.001; # compared as indicated; ^#^ *P* < 0.05; ^##^ *P* < 0.01; ^###^ *P* < 0.001; *ICAM* intercellular adhesion molecule, *SFO* subfornical organ, *LPS* lipopolysaccharide, *SHAM* sham surgery, *VGX* bilateral vagotomy, *VGX + VNS* bilateral vagotomy and distal vagus nerve stimulation

LPS induced the expression of CXCL1 and the ICAM-1 in the hypothalamus and cortex on mRNA level. In the hypothalamus, vagus nerve stimulation significantly reduced the increased expression of CXCL1 and ICAM-1 (Fig. 5), whereas the immunohistological results of all groups did not show any difference between the constitutively increased levels in the SFO/choroid plexus (Additional file 1B-E). In the cortex, vagotomy and vagus nerve stimulation resulted in a reduced expression of the LPS-induced CXCL1 and ICAM-1 mRNA expression (Fig. 5), which did not result in immunohistologically significant differences between the LPS-treated groups (Additional file 1H-J). However, LPS induced an increase in ICAM-1 immunoreactivity compared to the sham group in particular in small brain blood vessels.

### Vagus nerve stimulation reduces the number of neutrophil granulocytes, which are colocalized with ICAM-1

**Fig. 6:**
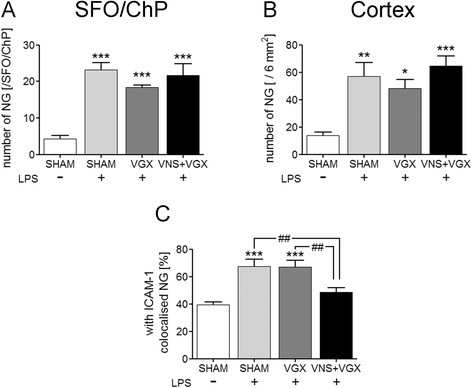
Immunohistochemical analysis of the number of neutrophil granulocytes in the SFO and neighboring choroid plexus (**a**) and the cortex (**b**) and their colocalization with ICAM-1 (**c**) 4.5 h after LPS or vehicle administration (*n* = 5). LPS increased the number of neutrophil granulocytes in the SFO and neighboring choroid plexus and the cortex as well as their colocalization with ICAM-1. There were no differences in the number of neutrophil granulocytes between the septic groups, but interestingly, vagus nerve stimulation reduced the percentage of neutrophil granulocytes that were colocalized with ICAM-1 compared to the LPS + SHAM group (*P* < 0.01) and the LPS + VGX group (*P* < 0.01). Data are given as means ± SEM. Significance is given as: * compared to SHAM; *P < 0.05; **P < 0.01; ***P < 0.001; # compared as indicated; ^#^ *P* < 0.05; ^##^ *P* < 0.01; ^###^ *P* < 0.001; *ICAM* intercellular adhesion molecule, *SFO* subfornical organ, *ChP* choroid plexus, *LPS* lipopolysaccharide, *NG* neutrophil granulocytes, *SHAM* sham surgery, *VGX* bilateral vagotomy, *VGX + VNS* bilateral vagotomy and distal vagus nerve stimulation

**Fig. 7:**
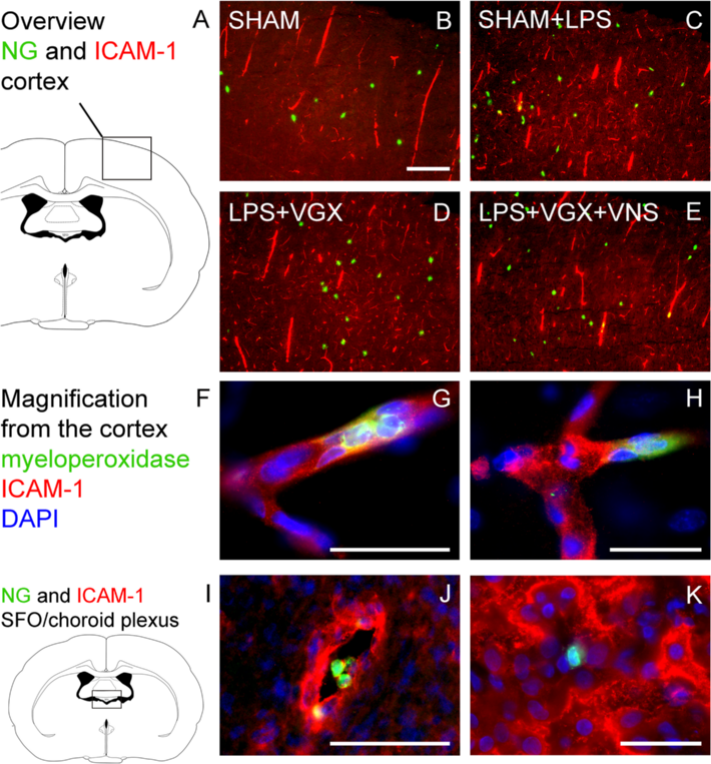
Immunohistochemical pictures of the colocalization of neutrophil granulocytes and ICAM-1 in the cortex (**a**–**e**) and magnifications from the cortex (**f**–**h**) and the SFO and neighboring choroid plexus (**i**–**k**). LPS increased the number of neutrophil granulocytes (*green*) that were colocalized with ICAM-1 (*red*) in the cortex which was prevented by vagus nerve stimulation (for quantification see Fig. 8). Pictures **g**–**h** and **j**–**k** show representative magnifications of this colocalization in the cortex and the SFO and neighboring choroid plexus of LPS-stimulated animals. Similar pictures could be observed in all the experimental groups. The scale bar in picture **b** represents 200 μm and applies to the pictures **b**–**e**. The *scale bar* in picture **g** and **h** represents 25 μm; the one in picture **j** 50 μm and the one in picture **k** 25 μm, respectively

Immunohistochemical analysis revealed that LPS increased the number of neutrophil granulocytes in the SFO and neighboring choroid plexus as well as in the cortex (Fig. 6 and Additional file 2). However, there were no significant differences in the number of neutrophil granulocytes between the septic groups. Additionally, LPS increased the percentage of neutrophil granulocytes that were colocalized with ICAM-1 in the cortex. Interestingly, this effect was prevented by vagus nerve stimulation (Figs. 6c and 7).

### Vagus nerve stimulation reduces leptin plasma concentration

**Fig. 8:**
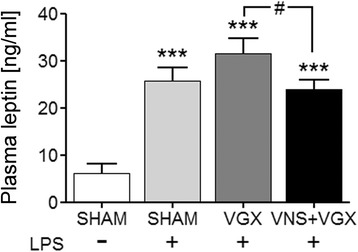
**a** Leptin plasma concentration 4.5 h after LPS or vehicle administration (*n* = 5 in the SHAM group and *n* = 8 in the septic groups). LPS increased leptin plasma levels, which were reduced by vagus nerve stimulation compared to the LPS + VGX group (*P* < 0.05)

LPS increased leptin plasma levels in all groups (Fig. 8). Vagal intervention resulted in significant differences (*P* < 0.5) with lower circulating leptin levels in the vagus nerve-stimulated group.

## Discussion

In our experiments, stimulation of the distal trunk of the vagus nerve showed anti-inflammatory properties, as reported by several other authors [11, 22–25], reducing peripheral cytokines in the current study. Additionally, VGX + VNS was beneficial for brain microcirculation and brain function, preventing the LPS-induced decline of the SEP and EFVR. These results are in line with our previous work [13] and indicate that VNS might be a valuable tool for the treatment of sepsis and sepsis-associated delirium. Still, the underlying mechanisms, especially concerning the effects of VNS on the brain, are not well understood. Therefore, several transcription factors and cytokines were measured in a region with complete blood-brain barrier (BBB) (cortex) and a region lacking in parts a tight BBB (hypothalamus) as markers for central inflammatory activation [26]. Interestingly, VGX + VNS caused a significant decrease of the IL-6, IL 10, NF-IL6, SOCS3, CXCL1, ICAM-1, and COX2 mRNA expression in the hypothalamus indicating an anti-inflammatory effect of efferent VNS in this region. In contrast to the hypothalamus, vagotomy independent of VNS reduced the mRNA expression of IL-1β, IkBα, SOCS3, NF-IL6, CXCL1, ICAM-1, and COX2 in the cortex pointing to an anti-inflammatory effect of VGX in the cortex. Here, we have to take into account the different properties of efferent and afferent vagus nerve stimulation as well as the different brain regions. While it was shown that efferent VNS has anti-inflammatory properties [11], afferent VNS signals can, for example, induce sickness behavior [27, 28]. In line with our current results, Layé et al. [10] and Hansen et al. [9] have previously shown that subdiaphragmatic vagotomy blunted the LPS- or IL-1β-induced IL-1β induction in the hippocampus and hypothalamus independent of peripheral IL-1β expression. In this context, it has been suggested that the anti-inflammatory effect in the brain of an interruption of vagal afferents depends on the route of administration and the dose of the systemically injected inflammatory stimulus [8]. For example, Bluthe et al. reported that sickness behavior induced by intraperitoneal (ip) but not intravenous (iv) injections of IL-1β could be antagonized by vagotomy [29]. It was argued that the iv-route of administration caused a much stronger activation of the humoral afferent route of immune-to-brain signaling, which could override the pro-inflammatory effects of the afferent part of the vagus nerve. Others, however, provided evidence that fever induced by iv injections of LPS could be attenuated by vagotomy ([8] for review). In an analogy, we found an anti-inflammatory effect of vagotomy in the brain independent from systemic effects of the vagal immune-modulation. One likely explanation might be that the BBB shields the cortex from a direct humoral or cellular inflammation from the blood stream. One prerequisite for this assumption is an intact BBB. In similar studies using the same animal model, we did not find hints for a breakdown of the BBB at the time point investigated [30]. In contrast, the hypothalamus contains regions that lack a tight BBB and, thus, are sensitive to direct systemic inflammatory factors. Systemic effects of anti-inflammatory vagus nerve stimulation, therefore, influence the local inflammatory response directly (Figs. 2 and 5).

Interestingly, the cerebral mRNA expression of TNFα did not differ between the sepsis groups although the TNFα plasma concentration was reduced by VNS. This apparent contradiction was recently clarified by Sun et al. [30]: nicotine induced the expression of the microRNA (miR)-124 via α7 nicotinic acetylcholine receptors (α7nAChR) on macrophages. The miR-124 inhibits the tumor necrosis factor-alpha converting enzyme (TACE) causing a reduced concentration of TNFα despite of an increased TNFα mRNA expression.

It is known that microcirculatory failure is a motor of organ dysfunction and microcirculatory changes precede the decline of brain function [31]. As VNS protects brain microcirculation, it seems likely that VNS may interfere with some mechanism regulating brain blood flow. One key factor in the regulation of cerebral blood flow is nitric oxide (NO). However, previous work showed that VNS does not influence the nitric oxide system in the brain [13] under septic conditions. Other important factors are prostaglandins which are linked to inflammation as well as regulation of the cerebral blood flow [32, 33]. In the literature, the influence of nicotinergic stimulation on the synthesis of PGE2 is controversially discussed. Stimulation of monocytes and microglia with nicotine caused in some studies an induction [34–36] of PGE2 while it had no influence [37, 38] or even lead to an inhibition [38, 39] of PGE2 synthesis in others. However, Le Maitre et al. [37] showed that PGE2 seems to influence the anti-inflammatory effects of VNS. In their study, mPGES-1 knockout animals were not able to develop a rise of acetylcholine in the spleen and a reduction of pro-inflammatory cytokines after VNS. Nevertheless, they were not able to detect an influence of VNS on the PGE2 expression in the periphery or the brain, so that the significance of PGE2 for the anti-inflammatory actions of VNS remained unclear. However, results of our current study point to an influence of VNS on PGE2 synthesis in the hypothalamus. Further analysis of arachidonic acid derivates could clarify this objective.

In addition to humoral factors, leukocytes are also thought to contribute to the pathogenesis of organ dysfunction during sepsis. While VNS did not influence microglial activation, our results suggest a modulatory effect of VNS on the migration of neutrophil granulocytes to the brain. In this regard, VNS reduced the mRNA expression of the neutrophil-specific chemokine CXCL1 and the adhesion molecule ICAM-1 in the hypothalamus, indicating a VNS-induced reduction of migration signals for neutrophil granulocytes. Interestingly, and possibly due to the early time point of investigation, protein-level immunohistochemical analysis did not show any differences of ICAM-1 between the septic groups neither in the SFO and neighboring choroid plexus nor in the cortex. This might be due to different regulatory processes on mRNA and protein level, which act in a time-dependent manner. Neutrophil granulocytes are thought to migrate into the brain parenchyma 12 to 24 h after an insult [40–45]. At the early time point (4.5 h) of this study, they were probably still adhering to and rolling on endothelial cells [45]. Interestingly, the number of neutrophil granulocytes counted in the cortex did not differ between the septic groups, but we could show here, for the first time, that VNS reduced the colocalization between ICAM-1 and neutrophil granulocytes indicating a decreased interaction and adhesion of neutrophil granulocytes and ICAM-1 on cerebral blood vessels. This might contribute to the beneficial effects of VNS on brain microcirculation. As ICAM-1 protein expression was not influenced by VNS, this might rather point to a VNS-induced modulation of neutrophil granulocytes themselves. Mechanism by which VNS modulates neutrophil granulocytes should be further analyzed in future studies. As one possible mechanism, we analyzed leptin plasma concentration, which was reduced by VNS. Leptin is well known as appetite-regulating hormone but recently its function as neuro-immune mediator became known [46]. In this regard, leptin is involved in the migration of neutrophil granulocytes to the brain [40, 44, 47]. Therefore, upon other in part mentioned mechanisms, VNS might also inhibit neutrophil migration to the brain in a leptin-dependent manner.

Another hint for the relevance of the cerebral microcirculation on neuronal function in the early inflammation stages is that the cortical anti-inflammatory effects of vagotomy neither protected from neurovascular dysfunction nor decline in evoked potential amplitudes. The matter of vagus nerve stimulation on the intact nerve, which most likely resembles clinical conditions using external nerve stimulators might be addressed in future studies. In this context, the possibly opposing effect of transcutaneous (clinical) stimulation of the intact vagus nerve on systemic inflammation and inflammatory modifications within the brain should be considered.

## Conclusions

We addressed for the first time the potential brain-related mechanisms of the protective effect of efferent vagus nerve stimulation on neurophysiological parameters, e.g., neurovascular coupling during an early phase of severe inflammation. Contributing factors pertain to (1) reduced interaction of neutrophil granulocytes with brain endothelial cells linked to reduced circulating leptin levels and (2) an attenuated inflammatory response of the brain as reflected by changes in several inflammatory markers including inflammatory transcriptions factors namely NF-IL6. (3) Moreover, new insights were revealed into the brain area-specific influences of vagus nerve stimulation/vagotomy depending on its access to circulating mediators, e.g., cortical areas compared to hypothalamic structures in close vicinity to circumventricular organs with a leaky blood-brain barrier.

## Abbreviations

BBB, blood-brain barrier; BP, blood pressure; ChP, choroid plexus; COX, cyclooxygenase; DAPI, 4.6-diamidino-2-phenylindole; DNA, deoxyribonucleic acid; dNTP, deoxynucleoside triphosphates; EFVR, evoked flow velocity response; ELANE, elastase, neutrophil expressed; ELISA, enzyme-linked immunosorbent assay; ICAM, intercellular adhesion molecule; IL, interleukin; ip, intraperitoneal; iv, intravenous; IkBα, inhibitor of kBα; LDF, laser-Doppler flowmetry; LPS, lipopolysaccharide; miR, microRNA; mPGES, microsomal prostaglandin E synthase; MPO, myeloperoxidase; MRI, magnetic resonance imaging; mRNA, messenger ribonucleic acid; N., nervus; Ncl., nucleus; NDS, normal donkey serum; NF-IL6, nuclear factor interleukin 6; NFkB, nuclear factor kappa B; NG, neutrophil granulocyte; NO, nitric oxide; PBS, phosphate-buffered saline; pCO_2_, partial pressure of carbon dioxide; PCR, polymerase chain reaction; PG, prostaglandin; pO_2_, partial pressure of oxygen; RNA, ribonucleic acid; SAD, sepsis-associated delirium; SD, standard deviation; SEM, standard error of the mean; SEP, somatosensory evoked potentials; SFO, subfornical organ; SHAM, sham surgery; SIRS, systemic inflammatory response syndrome; SOCS, suppressor of cytokine signaling; STAT, signal transducers and activators of transcription; TACE, tumor necrosis factor-alpha converting enzyme; TNF, tumor necrosis factor; VGX, bilateral vagotomy; VNS, distal vagus nerve stimulation; α7nAChR, α7 nicotinic acetylcholine receptor

## Additional files

Additional file: 1 Immunohistochemical analysis of ICAM-1 in the SFO and neighboring choroid plexus (A–E) and cortex (F–J) as well as two magnifications from the cortex (K–M) 4.5 h after LPS or vehicle administration (*n* = 5). In the SFO and neighboring choroid plexus there was a strong ICAM-1 immunoreactivity independent of the LPS administration or manipulation of the vagus nerve. In the cortex LPS increased the ICAM-1 immunoreactivity but there were still no apparent differences between the septic groups (for quantification see Fig. 6). Pictures (L) and (M) show a magnification of a representative ICAM-1-positive blood vessel in the cortex of a LPS + VGX animal which could be observed in similar ways in the other experimental groups. ICAM-1 (intercellular adhesion molecule) is indicated as white signal on a black background. SFO, subfornical organ; LPS, lipopolysaccharide; SHAM, sham surgery; VGX, bilateral vagotomy; VGX + VNS, bilateral vagotomy and distal vagus nerve stimulation. (PNG 3031 kb) Additional file: 2 Immunohistochemical pictures of neutrophil granulocytes in the SFO and neighboring choroid plexus (A–E) and the cortex (F–K) as well as two magnifications from the cortex (L–M) 4.5 h after LPS or vehicle administration (*n* = 5). LPS caused an increase of the number of neutrophil granulocytes in the SFO and neighboring choroid plexus and the cortex that did not differ between the septic groups (for quantification see Fig. 8). Pictures (L) and (M) show magnifications of representative neutrophil granulocytes in LPS-treated animals in the cortex (L) and the SFO (M). Myeloperoxidase as marker of neutrophil granulocytes is presented in green (B–E and L–M) or as white dots (G–K), nuclei are stained in blue and ICAM-1 (intercellular adhesion molecule) in red. SFO, subfornical organ; LPS, lipopolysaccharide; SHAM, sham surgery; VGX, bilateral vagotomy; VGX + VNS, bilateral vagotomy and distal vagus nerve stimulation. (PNG 1870 kb)
