# Supplementary figures and images for: Modulatory effects of vagal stimulation on neurophysiological parameters and the cellular immune response in the rat brain during systemic inflammation
Source: Intensive Care Med Exp. 2016 Jun 29;4:19. doi: 10.1186/s40635-016-0091-4 (PMC4927529; doi:10.1186/s40635-016-0091-4)

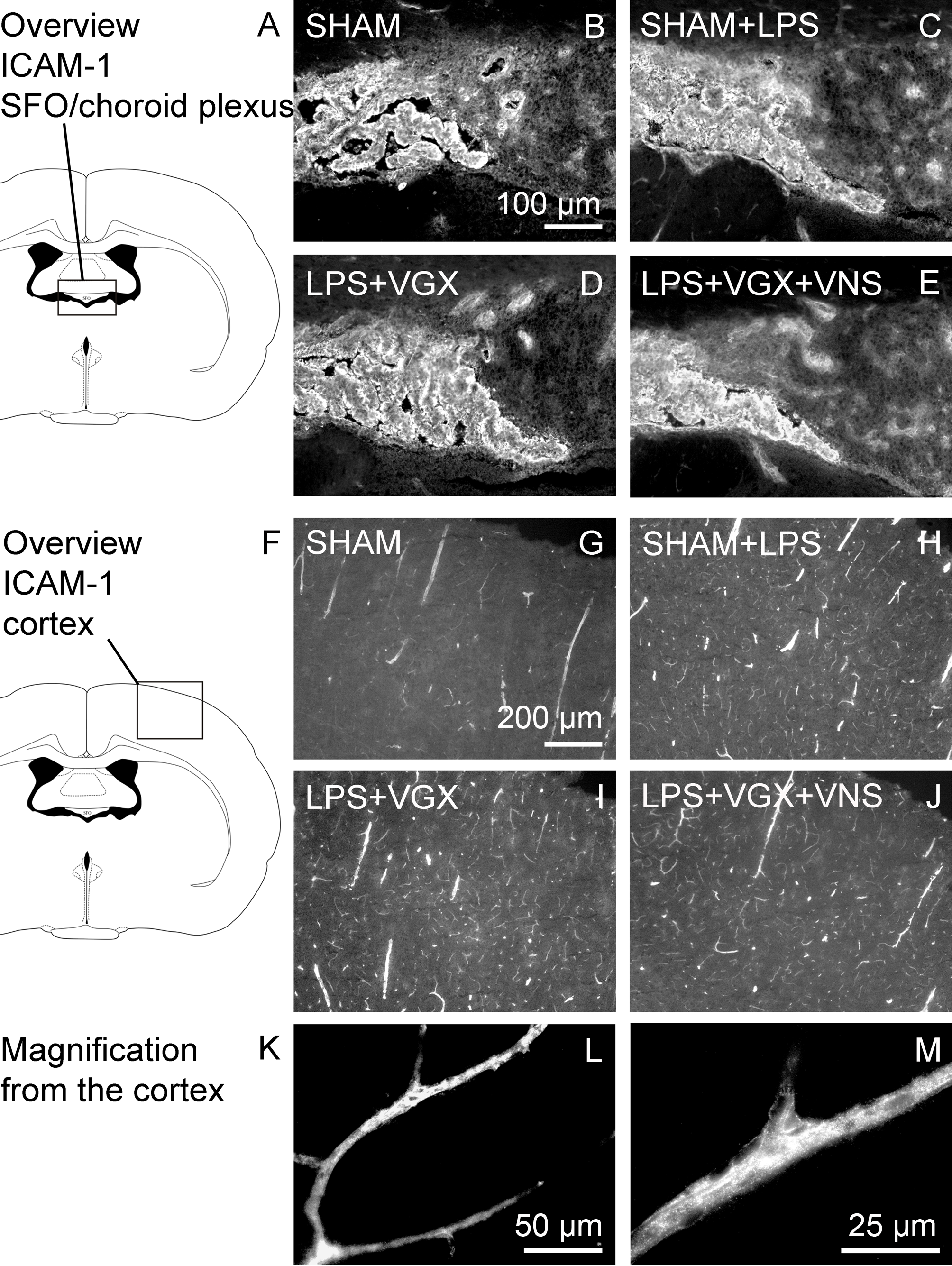

Supplement: Additional file: 1 — Immunohistochemical analysis of ICAM-1 in the SFO and neighboring choroid plexus (A–E) and cortex (F–J) as well as two magnifications from the cortex (K–M) 4.5 h after LPS or vehicle administration (n = 5). In the SFO and neighboring choroid plexus there was a strong ICAM-1 immunoreactivity independent of the LPS administration or manipulation of the vagus nerve. In the cortex LPS increased the ICAM-1 immunoreactivity but there were still no apparent differences between the septic groups (for quantification see Fig. 6). Pictures (L) and (M) show a magnification of a representative ICAM-1-positive blood vessel in the cortex of a LPS + VGX animal which could be observed in similar ways in the other experimental groups. ICAM-1 (intercellular adhesion molecule) is indicated as white signal on a black background. SFO, subfornical organ; LPS, lipopolysaccharide; SHAM, sham surgery; VGX, bilateral vagotomy; VGX + VNS, bilateral vagotomy and distal vagus nerve stimulation. (PNG 3031 kb) [file 40635_2016_91_MOESM1_ESM.png]

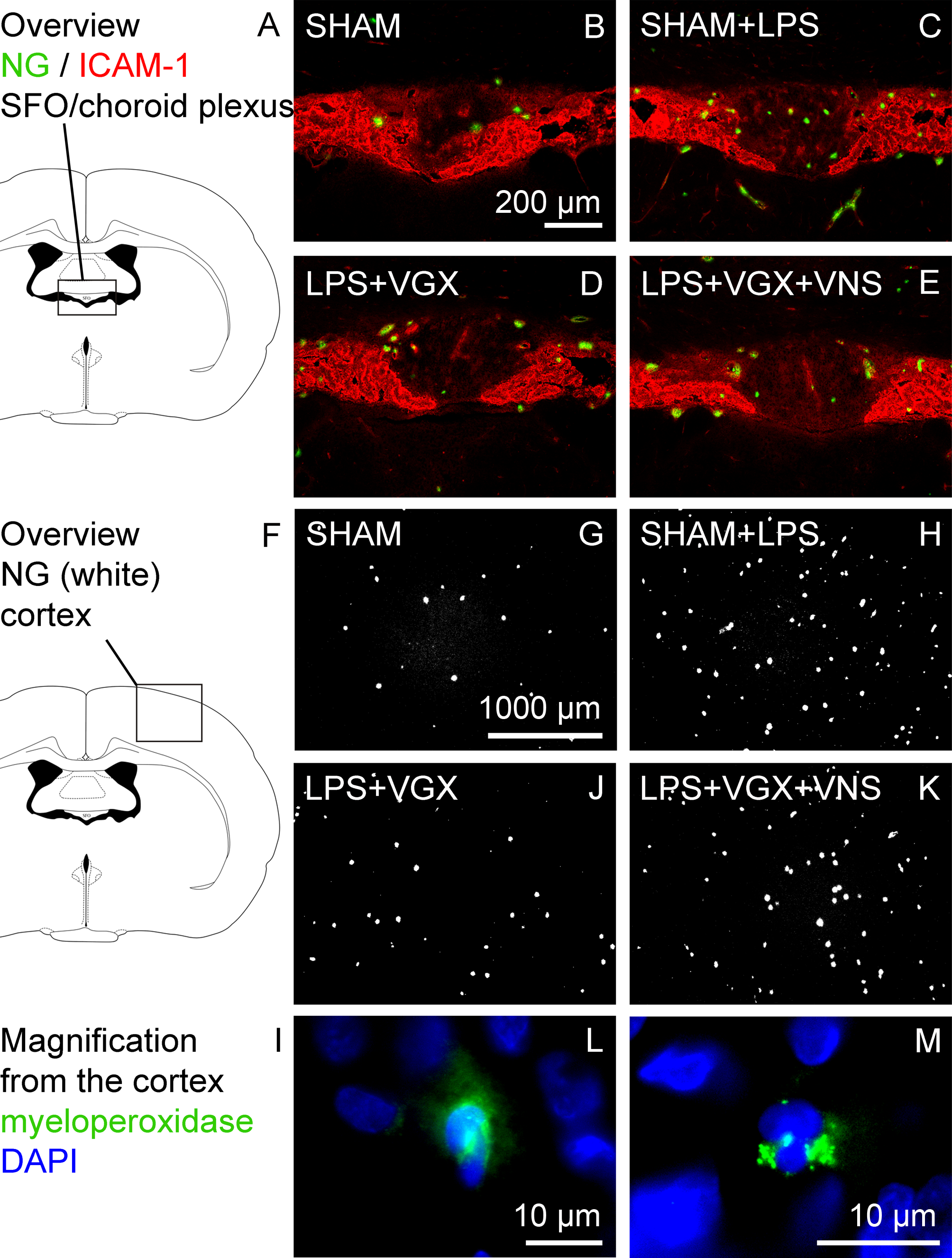

Supplement: Additional file: 2 — Immunohistochemical pictures of neutrophil granulocytes in the SFO and neighboring choroid plexus (A–E) and the cortex (F–K) as well as two magnifications from the cortex (L–M) 4.5 h after LPS or vehicle administration (n = 5). LPS caused an increase of the number of neutrophil granulocytes in the SFO and neighboring choroid plexus and the cortex that did not differ between the septic groups (for quantification see Fig. 8). Pictures (L) and (M) show magnifications of representative neutrophil granulocytes in LPS-treated animals in the cortex (L) and the SFO (M). Myeloperoxidase as marker of neutrophil granulocytes is presented in green (B–E and L–M) or as white dots (G–K), nuclei are stained in blue and ICAM-1 (intercellular adhesion molecule) in red. SFO, subfornical organ; LPS, lipopolysaccharide; SHAM, sham surgery; VGX, bilateral vagotomy; VGX + VNS, bilateral vagotomy and distal vagus nerve stimulation. (PNG 1870 kb) [file 40635_2016_91_MOESM2_ESM.png]
